# Elucidating Under-Studied Aspects of the Link Between Obesity and Multiple Myeloma: Weight Pattern, Body Shape Trajectory, and Body Fat Distribution

**DOI:** 10.1093/jncics/pkz044

**Published:** 2019-06-24

**Authors:** Catherine R Marinac, Catherine A Suppan, Edward Giovannucci, Mingyang Song, Ane S Kværner, Mary K Townsend, Bernard A Rosner, Timothy R Rebbeck, Graham A Colditz, Brenda M Birmann

**Affiliations:** 1 Division of Population Sciences, Department of Medical Oncology, Dana-Farber Cancer Institute, Boston, MA; 2 Department of Epidemiology, Harvard T.H. Chan School of Public Health, Boston, MA; 3 Department of Nutrition, Harvard T.H. School of Public Health, Boston, MA; 4 Channing Division of Network Medicine, Department of Medicine, Brigham and Women’s Hospital and Harvard Medical School, Boston, MA; 5 Clinical and Translational Epidemiology Unit and Division of Gastroenterology, Massachusetts General Hospital, Boston, MA; 6 Department of Nutrition, Institute of Basic Medical Sciences, University of Oslo, Oslo, Norway; 7 Department of Cancer Epidemiology, Moffitt Cancer Center, Tampa, FL; 8 Department of Surgery and Alvin J. Siteman Cancer Center, Washington University School of Medicine, and Barnes Jewish Hospital, St. Louis, MO

## Abstract

**Background:**

Although obesity is an established modifiable risk factor for multiple myeloma (MM), several nuanced aspects of its relation to MM remain unelucidated, limiting public health and prevention messages.

**Methods:**

We analyzed prospective data from the Nurses’ Health Study and Health Professionals Follow-Up Study to examine MM risk associated with 20-year weight patterns in adulthood, body shape trajectory from ages 5 to 60 years, and body fat distribution. For each aforementioned risk factor, we report hazard ratios (HRs) and 95% confidence intervals (CIs) for incident MM from multivariable Cox proportional-hazards models.

**Results:**

We documented 582 incident MM cases during 4 280 712 person-years of follow-up. Persons who exhibited extreme weight cycling, for example, those with net weight gain and one or more episodes of intentional loss of at least 20 pounds or whose cumulative intentional weight loss exceeded net weight loss with at least one episode of intentional loss of 20 pounds or more had an increased MM risk compared with individuals who maintained their weight (HR = 1.71, 95% CI = 1.05 to 2.80); the association was statistically nonsignificant after adjustment for body mass index. We identified four body shape trajectories: lean-stable, lean-increase, medium-stable, and medium-increase. MM risk was higher in the medium-increase group than in the lean-stable group (HR = 1.62, 95% CI = 1.22 to 2.14). Additionally, MM risk increased with increasing hip circumference (HR per 1-inch increase: 1.03, 95% CI = 1.01 to 1.06) but was not associated with other body fat distribution measures.

**Conclusions:**

Maintaining a lean and stable weight throughout life may provide the strongest benefit in terms of MM prevention.

Multiple myeloma (MM) is a malignant plasma cell neoplasm and the second most common hematologic malignancy in the United States (1). Advancements in MM treatments have dramatically improved the median survival time in patients in recent decades; however, these treatments are associated with financial toxicity (2) and do not achieve cure. These factors, coupled with the increasing incidence of MM observed for several decades (3), underscore the importance of identifying MM-prevention strategies.

Although improvements in case ascertainment may explain some of the increase in MM incidence, a parallel rise in the prevalence of obesity may have contributed (4). Obesity, which results in inflammation and deregulation of numerous endogenous growth factors important to MM pathogenesis, is the only known modifiable risk factor both for MM and its precursor condition, monoclonal gammopathy of unknown significance (5–7)—which precedes all MM cases (8)—and is associated with an increased risk of malignant transformation of monoclonal gammopathy of unknown significance to MM (9). Despite the well-established links between obesity and various stages of myelomagenesis, several nuanced aspects of this association remain unclear, including whether intentional weight loss or a specific weight pattern in adulthood confers protection against MM and whether the influence of obesity on MM risk varies over the life course, because some but not all studies have reported the greatest MM risk in persons overweight or obese both in younger and later adulthood (10–13). It is also unclear whether body fat distribution influences disease risk independently of overall adiposity (13–16).

We evaluated the association of 20-year weight patterns in adulthood, trajectory of body shape through age 60 years, and measures of central and peripheral adiposity with MM risk using data from two large cohorts. Our objective was to confirm and expand on previous investigations of these exposures (11,13–18) with a larger number of cases and longer duration of follow-up (than most)—as well as with a novel life course approach—to optimize strategies regarding obesity and MM prevention.

## Methods

### Participants

We conducted this prospective analysis in the Nurses’ Health Study (NHS) and the Health Professionals Follow-Up Study (HPFS) among participants with no baseline history of cancer (except nonmelanoma skin cancer). The NHS was established in 1976 when 121 700 female US registered nurses ages 30–55 years returned the enrollment questionnaire (19). The HPFS enrolled 51 529 US licensed male health professionals ages 40–75 years in 1986. Participants in both cohorts have completed follow-up questionnaires biennially since enrollment to update lifestyle and disease history information (20,21). The study protocol was approved by the institutional review boards of the Brigham and Women’s Hospital and Harvard T.H. Chan School of Public Health and those of participating registries as required. Informed consent was implied by return of the questionnaires.

### Measures

#### 

##### 

***Weight, Height, and Weight Patterns***. Participants have reported current weight biennially since enrollment and provided height at enrollment and young adult weight in 1980 (NHS, for age 18 years) or 1986 (HPFS, for age 21 years). In 1992, participants in both cohorts answered the question, “Within the last 20 years, how many times did you lose each of the following amounts of weight on purpose (excluding illness [or pregnancy])?” with response categories of 5–9, 10–19, 20–49, and 50 or more pounds. Additionally, we estimated long-term net weight change as the difference in self-reported weights between 1976 and 1992 in NHS and 1981 and 1992 in HPFS. Self-reported weights were highly correlated with measured weights (correlations of 0.97) both in men and women (22). We combined the data on intentional weight loss and net weight changes (23) to define six 20-year weight pattern categories (“weight patterns”): weight loss, weight maintenance, weight gain, light cycling, medium cycling, and extreme cycling (defined in Table 1).

**Table 1.** Weight patterns during a 20-year period (1972–1992) in the NHS and HPFS*

Category	Description
Weight loss	Intentional weight loss between 1972 and 1992 with no net weight gain of ≥5 pounds over similar period
Weight maintenance	No reported episodes of intentional weight loss between 1972 and 1992, with no net weight loss or gain ≥5 pounds over similar period
Weight gain	No reported intentional weight loss between 1972 and 1992, with net weight gain of ≥5 pounds over similar period
Light cycling	Intentional weight loss between 1972 and 1992 with a maximum weight loss of 5 to 9 pounds per episode and net weight gain ≥5 pounds over a similar period or a cumulative reported intentional loss exceeding net weight loss
Medium cycling	Intentional weight loss between 1972 and 1992 with a maximum weight loss of 10 to 19 pounds per episode and net weight gain ≥5 pounds over a similar period or a cumulative reported intentional loss exceeding net weight loss
Extreme cycling	Intentional weight loss between 1972 and 1992 with a maximum weight loss of ≥20- pounds per episode and net weight gain ≥5 pounds over a similar period or a cumulative reported intentional loss exceeding net weight loss

* Net weight loss or gain was calculated as weight changes between 1976 and 1992 in NHS, and between 1981 and 1992 in HPFS. HPFS = Health Professionals Follow-Up Study; NHS = Nurses’ Health Study.

##### 

***Body Shape***. In 1988, participants recalled their body shape at ages 5, 10, 20, 30, and 40 years by selecting the “somatotype” pictogram that best represented their shape among nine choices with increasing body fatness (24). This assessment of earlier life body shape was validated in a study of 181 participants in the Third Harvard Growth Study; the correlations between recalled body shape and body mass index (BMI) measured at approximately the same ages ranged from 0.53 to 0.75 for all ages, except the correlation for male participants at age 5 years (0.36) (25).

Body shape data were collected only through age 40 years, so we used self-reported height and current weight to calculate BMI at ages 45, 50, 55, and 60 years. We then converted those BMIs to the same nine-level scale as the somatotype variables, as previously described (26). The cut-points for the nine-level variable categories were calculated as the median BMI of the given somatotype at age 40 years, plus a constant to account for weight gain between the corresponding ages (see Supplementary Methods, available online) (26).

##### 

***Waist and Hip Circumference***. Waist and hip circumference were self-reported for the first time in 1987 (HPFS) and 1986 (NHS), with updates in 1996 and 2008 (HPFS) or 2000 (NHS). The self-reported waist and hip circumference measures were validated against technician measurements, with correlation coefficients ranging from 0.84 to 0.95 (22).

##### 

***Outcome Ascertainment***. Diagnoses of MM were identified on study questionnaires or (for a small proportion) when confirming vital status. We then sought written consent for medical record review; trained personnel blinded to exposure status reviewed medical records to confirm the occurrence and date of MM diagnosis. When medical records were unavailable, we pursued case confirmation via linkage to state tumor registries. Deaths were identified by next of kin, the postal service, or routine searches of the National Death Index, which was shown to be sensitive and specific in these cohorts (27,28).

### Statistical Analysis

Primary exposures included weight patterns, body shape trajectory from age 5 through 60 years, waist and hip circumference, and the waist-to-hip ratio (WHR). Weight pattern variables were modeled categorically; waist and hip circumference and the WHR were modeled using cohort (ie, sex)-specific quartiles and as continuous variables. Body shape trajectories were identified separately in each cohort via a group-based modeling approach and implemented using SAS Proc Traj (29), which identified subgroups within each cohort that share a similar underlying trajectory of body shape through age 60 years, as described previously (26). Briefly, the longitudinal body shape data were fitted by maximum likelihood methods as a mixture of multiple latent trajectories in a censored normal model with a polynomial function of the time scale (age) (26). To optimize trajectory classification, we included individuals with at least three longitudinal body shape variables available. The Bayesian Information Criterion, average posterior probability of class assignment, odds of correct classification, and considerations of case and sample size distributions were used to identify the number of trajectories that best fit the data (30). Models with four trajectories and a cubic function of age demonstrated adequate fit in each cohort based on these parameters; we named trajectories per the visual pattern of change in body shape with age. We assigned individuals to the trajectory for which their posterior probability of membership was highest.

Person-time was calculated from enrollment—or, for analyses of waist, hip, and the WHR in NHS, from 1986—to the earliest among dates of diagnosis of MM, another cancer (except non-melanoma skin cancer), death, or January (HPFS) or June (NHS) 2014. In Cox proportional-hazards models stratified by age (months) and calendar period of follow-up, we calculated hazard ratios (HRs) and 95% confidence intervals (CIs) for the association of a given exposure category or continuous measure with the risk of MM. Initial models of weight patterns were adjusted for height. To investigate the relative contribution of BMI to the observed weight pattern associations, we adjusted separate models for cumulative average BMI (10) instead of height; additional exploratory models were adjusted for weight or BMI at different time points (eg, young adulthood), or change in weight between younger and later adulthood (10). We adjusted models of body shape trajectories for height and ran models of waist and hip circumference and WHR with and without adjustment for current BMI. The assumption of proportionality was verified using interactions between the exposure of interest and the (log-)time scale.

To facilitate cross-study comparisons, body shape trajectories and corresponding Cox models were rerun using only ages 20 to 60 years. In sensitivity analyses, for body shape-MM associations, we excluded individuals diagnosed with MM before age 60. For weight pattern-MM associations, we excluded individuals diagnosed before 1992 (ie, when the exposure was queried) to assess the potential influence of bias. Exclusions did not materially alter the effect estimates; thus, we retained all cases in final analyses to maximize power. We first ran all models separately by cohort and tested for heterogeneity by cohort (sex) using a fixed-effects meta-analysis. We found no evidence of statistically significant heterogeneity (all *P* values for heterogeneity ≥ .17 except the one for weight loss [*P* = .04]) and therefore ran all final models on a pooled sample with adjustment for sex.

## Results

We identified 582 incident primary diagnoses of MM (n = 354 in the NHS and n = 228 in the HPFS) during 4 280 712 person-years of follow-up. Participants had a mean age of 46.3 years (SD = 9.7) and a mean BMI of 24.3 kg/m^2^ (SD = 4.0) at enrollment. Weight cycling, the most common 20-year weight pattern identified, occurred in 75.3% of the cohort members (Supplementary Table 1, available online). Weight maintainers comprised only 7.2% of the cohort; 3.8% lost weight without regain.

We observed a positive association between weight cycling and MM risk for all weight cycling categories, and in particular for individuals who exhibited extreme weight cycling (Table 2). These individuals had a statistically significantly increased risk of MM compared with weight maintainers (HR = 1.71, 95% CI = 1.05 to 2.80). The association between extreme weight cycling and MM risk was statistically nonsignificant after adjustment for cumulative average BMI (HR = 1.36, 95% CI = 0.81 to 2.30). Adjustment for young adult BMI instead of cumulative average BMI also resulted in a statistically nonsignificant hazard ratio, albeit with less attenuation (HR = 1.57, 95% CI = 0.94 to 2.63), as did adjusting instead for weight change between younger and later adulthood. The increased MM risk among weight cyclers did not appear restricted to a specific BMI stratum (Supplementary Table 2, available online); however, we were limited to looking at only two categories both of weight cycling and BMI because of our relatively small case count.

**Table 2.** 20-Year weight patterns and the risk of MM among participants in the NHS and HPFS

	NHS			HPFS			Pooled*^,^†
Model	Cases	Person-years	HR (95% CI)‡	Cases	Person-years	HR (95% CI)‡	HR (95% CI)‡
Model 1: Adjusted for height							
Weight loss	2	61 486	0.37 (0.08 to 1.71)	11	31 727	2.22 (0.96 to 5.12)	1.30 (0.65 to 2.62)
Weight maintenance	9	115 066	1	12	78 065	1	1
Weight gain	34	325 182	1.51 (0.72 to 3.15)	15	85 539	1.35 (0.62 to 2.92)	1.52 (0.90 to 2.55)
Light cycling	67	621 402	1.47 (0.73 to 2.97)	30	166 543	1.40 (0.71 to 2.78)	1.54 (0.95 to 2.49)
Medium cycling	46	607 013	1.10 (0.54 to 2.27)	26	156 657	1.47 (0.73 to 2.96)	1.25 (0.76 to 2.05)
Extreme cycling	59	619 470	1.46 (0.72 to 2.97)	27	111 017	2.22 (1.11 to 4.45)	1.71 (1.05 to 2.80)
Model 2: Adjusted for cumulative average BMI							
Weight loss	2	61 486	0.37 (0.08 to 1.71)	11	31 727	2.20 (0.96 to 5.08)	1.31 (0.65 to 2.62)
Weight maintenance	9	115 066	1	12	78 065	1	1
Weight gain	34	325 182	1.42 (0.67 to 2.98)	15	85 539	1.27 (0.59 to 2.76)	1.44 (0.86 to 2.43)
Light cycling	67	621 402	1.35 (0.67 to 2.74)	30	166 543	1.32 (0.66 to 2.63)	1.43 (0.88 to 2.33)
Medium cycling	46	607 013	0.95 (0.46 to 1.99)	26	156 657	1.30 (0.64 to 2.64)	1.10 (0.66 to 1.82)
Extreme cycling	59	619 470	1.14 (0.53 to 2.42)	27	111 017	1.79 (0.86 to 3.74)	1.36 (0.81 to 2.30)

* *P* value for heterogeneity by sex (cohort) was *P*  equal to .04 for weight loss and *P*  equal to or greater than .40 for all other risk factors. BMI = body mass index; CI = confidence interval; HPFS = Health Professionals Follow-Up Study; HR = hazard ratio; MM = multiple myeloma; NHS = Nurses’ Health Study.

† Pooled models additionally adjusted for sex (cohort).

‡ Hazard ratios and 95% confidence intervals were calculated in Cox proportional-hazards models stratified on age and calendar period of follow-up.

**Figure 1.**
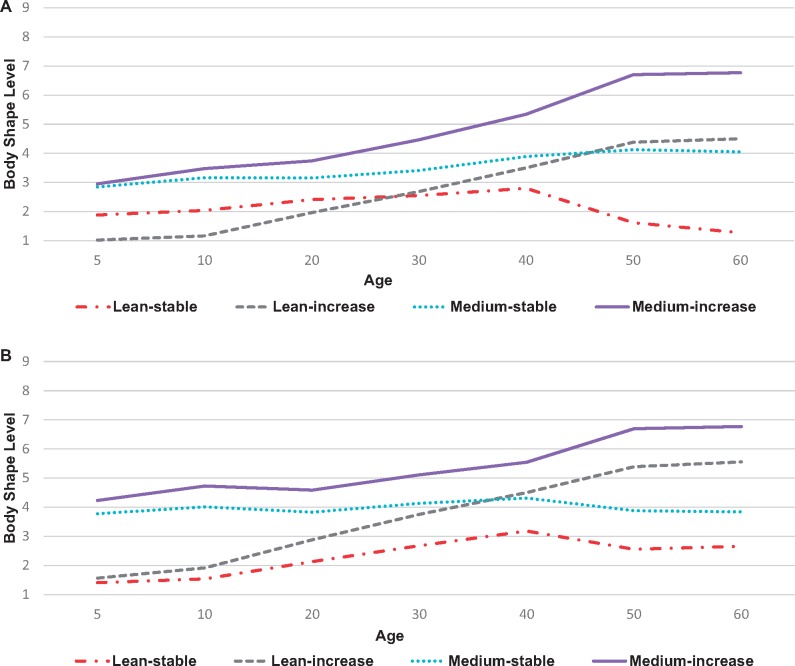
Trajectories of body shape across the life span among participants in the Nurses’ Health Study (NHS) and Health Professionals Follow-Up Study (HPFS). A larger body shape level corresponds to greater body fatness. **A**) Trajectories for women in the NHS. **B**) Trajectories for men in the HPFS.

We identified four roughly evenly distributed latent trajectories of body shape from age 5 to 60 years: one featuring a lean body shape throughout (“lean-stable”; 20% of sample), one beginning lean and getting larger (“lean-increase”; 28%), one beginning at a midrange shape and remaining stable (“medium-stable”; 29%), and one beginning midrange and getting larger (“medium-increase”; 23%; Figure 1, A and B). The average posterior probability (0.85–0.97) and odds of correct classification (12.4–121.2) were high for each trajectory. The visual patterns of the trajectories corresponded to trends observed for BMI at different ages (Supplementary Table 3, available online). For example, mean young adult BMI for individuals in the lean-stable trajectory was 19.8 kg/m^2^ for women and 20.9 kg/m^2^ for men and stayed relatively stable across ages. In contrast, mean BMI for individuals in the medium-increase category was 23.7 kg/m^2^ (women) and 25.8 kg/m^2^ (men) and increased with age.

In body shape trajectory–related models, individuals with a medium-increase trajectory had a greater risk of MM compared with individuals in the lean-stable trajectory category (Table 3). After adjustment for height, participants in the medium-increase category had a 62% greater risk of MM compared with those in the lean-stable category (HR = 1.62, 95% CI = 1.22 to 2.14). When we excluded childhood and adolescent somatotypes, a “large-increase” category emerged and had a slightly stronger association with MM risk than the “medium-increase” category in the model that included all somatotypes (Supplementary Table 1; Supplementary Figure 1, A and B, available online). When modeled individually, childhood and adolescent somatotypes were not statistically significantly associated with future MM risk, whereas younger and middle adulthood somatotypes showed stronger positive associations (Supplementary Table 5, available online).

**Table 3.** Body shape trajectories and the risk of MM among participants in the NHS and HPFS

	NHS			HPFS			Pooled*^,^†
Category	Cases	Person-years	HR (95% CI)‡	Cases	Person-years	HR (95% CI)‡	HR (95% CI)‡
Lean-stable	58	673 466	1	30	182 785	1	1
Lean-increase	71	799 581	1.06 (0.75 to 1.50)	72	352 591	1.36 (0.89 to 2.10)	1.17 (0.89 to 1.53)
Medium-stable	104	1 093 971	1.20 (0.87 to 1.65)	38	181 441	1.39 (0.85 to 2.26)	1.27 (0.97 to 1.66)
Medium-increase	86	829 720	1.51 (1.08 to 2.12)	36	167 157	1.90 (1.16 to 3.13)	1.62 (1.22 to 2.14)

* *P* values for heterogeneity by sex (cohort) were all at least .37. CI = confidence interval; HPFS = Health Professionals Follow-Up Study; HR = hazard ratio; MM = multiple myeloma; NHS = Nurses’ Health Study.

† Pooled models were additionally adjusted for sex (cohort).

‡ Hazard ratios and 95% confidence intervals were calculated in Cox proportional-hazards models stratified by age and calendar period of follow-up and adjusted for height.

In models investigating body fat distribution variables, we observed a modest positive association between hip circumference and MM risk (height adjusted HR = 1.03, 95% CI = 1.01 to 1.06), which did not attenuate after adjustment for BMI (Table 4). Waist circumference and WHR were not associated with MM risk.

**Table 4.** Associations between central adiposity in adulthood, assessed using waist and hip circumference, and the risk of MM among participants in the NHS and HPFS

Model	NHS			HPFS			Pooled*^,^†
	Cases	Person-years	HR (95% CI)‡	Cases	Person-years	HR (95% CI)‡	HR (95% CI)‡
Hip circumference (inches)							
Model 1: adjusted for height							
Quartile 1	50	370 629	1	28	182 861	1	1
Quartile 2	29	275 588	0.81 (0.50 to 1.32)	47	198 344	1.51 (0.94 to 2.45)	1.19 (0.86 to 1.65)
Quartile 3	50	337 281	1.00 (0.65 to 1.54)	37	160 147	1.45 (0.87 to 2.42)	1.26 (0.91 to 1.74)
Quartile 4	48	286 663	1.07 (0.69 to 1.67)	37	152 593	1.53 (0.91 to 2.57)	1.33 (0.96 to 1.84)
HR per inch increase			1.02 (0.98 to 1.05)			1.06 (1.01 to 1.11)	1.03 (1.01 to 1.06)
Model 2: adjusted for current BMI							
Quartile 1	50	370 629	1	28	182 861	1	1
Quartile 2	29	275 588	0.85 (0.52 to 1.38)	47	198 344	1.37 (0.85 to 2.22)	1.18 (0.85 to 1.64)
Quartile 3	50	337 281	1.09 (0.69 to 1.72)	37	160 147	1.19 (0.70 to 2.03)	1.24 (0.89 to 1.73)
Quartile 4	48	286 663	1.21 (0.69 to 2.10)	37	152 593	1.02 (0.55 to 1.88)	1.24 (0.84 to 1.83)
HR per inch increase			1.03 (0.98 to 1.08)			1.02 (0.95 to 1.09)	1.03 (1.00 to 1.07)
Waist circumference (inches)							
Model 1: adjusted for height							
Quartile 1	35	319 916	1	26	181 535	1	1
Quartile 2	36	322 279	0.86 (0.54 to 1.38)	47	208 505	1.39 (0.85 to 2.27)	1.07 (0.76 to 1.49)
Quartile 3	53	320 735	1.06 (0.68 to 1.64)	33	139 584	1.35 (0.80 to 2.29)	1.18 (0.84 to 1.65)
Quartile 4	55	313 632	1.03 (0.66 to 1.61)	45	165 698	1.49 (0.89 to 2.48)	1.20 (0.86 to 1.66)
HR per inch increase			1.01 (0.97 to 1.04)			1.04 (1.00 to 1.08)	1.02 (0.99 to 1.04)
Model 2: adjusted for current BMI							
Quartile 1	35	319 916	1	26	181 535	1	1
Quartile 2	36	322 279	0.88 (0.55 to 1.42)	47	208 505	1.24 (0.76 to 2.04)	1.05 (0.75 to 1.47)
Quartile 3	53	320 735	1.09 (0.68 to 1.73)	33	139 584	1.11 (0.64 to 1.92)	1.13 (0.79 to 1.61)
Quartile 4	55	313 632	1.06 (0.62 to 1.80)	45	165 698	0.99 (0.53 to 1.84)	1.07 (0.72 to 1.58)
HR per inch increase			1.01 (0.97 to 1.04)			1.00 (0.95 to 1.06)	1.01 (0.97 to 1.04)
Waist-to-hip ratio							
Model 1: adjusted for height							
Quartile 1	27	318 006	1	46	218 485	1	1
Quartile 2	51	321 704	1.59 (0.99 to 2.55)	28	158 380	1.05 (0.62 to 1.79)	1.22 (0.88 to 1.70)
Quartile 3	53	319 018	1.49 (0.93 to 2.38)	35	149 401	1.22 (0.73 to 2.02)	1.23 (0.89 to 1.70)
Quartile 4	46	308 802	1.08 (0.66 to 1.76)	40	166 414	1.05 (0.67 to 1.63)	0.99 (0.72 to 1.36)
HR per 0.1 unit increase			0.93 (0.77 to 1.12)			0.95 (0.74 to 1.23)	0.93 (0.80 to 1.08)
Model 2: adjusted for current BMI							
Quartile 1	27	318 006	1	46	218 485	1	1
Quartile 2	51	321 704	1.59 (0.99 to 2.54)	28	158 380	1.01 (0.59 to 1.72)	1.20 (0.86 to 1.67)
Quartile 3	53	319 018	1.48 (0.92 to 2.37)	35	149 401	1.11 (0.67 to 1.85)	1.18 (0.85 to 1.63)
Quartile 4	46	308 802	1.06 (0.64 to 1.75)	40	166 414	0.87 (0.55 to 1.39)	0.92 (0.66 to 1.27)
HR per 0.1-unit increase			0.91 (0.75 to 1.11)			0.85 (0.65 to 1.11)	0.89 (0.76 to 1.04)

* *P* values for heterogeneity by sex (cohort) were all at least .17. Quartiles are sex specific and men and women with implausibly low measurements ( <29 inches for men, <20 inches for women) were excluded from relevant analyses. BMI = body mass index; CI = confidence interval; HPFS = Health Professionals Follow-Up Study; HR = hazard ratio; MM = multiple myeloma; NHS = Nurses’ Health Study.

† Pooled models additionally adjusted for cohort (sex).

‡ Hazard ratios and 95% confidence intervals were calculated in Cox proportional-hazards models stratified by age and calendar period of follow-up.

## Discussion

We undertook this analysis to elucidate the influence of obesity on MM risk by examining associations of weight patterns, body shape trajectory, and body fat distribution. We found that individuals who exhibited extreme weight cycling during a 20-year period had a greater risk of MM compared with individuals who maintained their weight. This increased risk of MM among weight cyclers was slightly attenuated after adjustment for BMI, suggesting that weight cycling may contribute to MM risk, in part, through its contribution to long-term weight. MM risk was also elevated among individuals with a larger and increasing body shape through age 60 years, compared with individuals who maintained a lean shape, as well as among individuals with a larger hip circumference.

A report from an International Agency for Research on Cancer working group found sufficient evidence for a causal effect of obesity on MM risk (6). The group’s recommendation was to avoid excess body fatness for MM prevention, although they concluded that a causal cancer-preventive effect of weight loss remains “to be established.” For individuals at risk for MM, an important question remains: Could weight loss reduce the risk of developing MM? The current study could not address that question directly given the small proportion of individuals with sustained weight loss. Instead, a majority of weight loss attempts in our study sample resulted in weight cycling, which has been associated with morbidity and early mortality (31) and which we have identified as a possible MM risk factor. The excess MM risk in the weight cycling group is mechanistically plausible, because weight cycling has been shown to negatively affect immune function through the reduction of natural killer cell cytotoxicity (32), thereby dampening an individual’s ability to survey and control tumor initiation. Weight cycling may also contribute to MM risk through its contribution to long-term weight. These data highlight the challenges in sustaining long-term weight loss and suggest a need for additional strategies to reduce MM risk for those who are already obese. To this end, metformin and aspirin, which can modulate some downstream effects of obesity, warrant investigation as alternatives (33–35).

A question remains regarding whether a specific age of exposure to obesity predisposes an individual to MM. We have previously identified early adult BMI as a MM risk factor with a stronger association than BMI in later adulthood (10), but few studies have examined whether measures of body fatness in childhood contribute to future risk. In the current study, although we did not observe a strong separate influence of childhood or adolescent body shape, our trajectory analysis suggests that individuals whose body shape is relatively large at younger ages and increases through age 60 years have the greatest risk of MM. Notably, this association between a large and increasing body size and MM risk was only slightly stronger when we excluded individuals younger than 20 years. These observations support the idea that the pathogenic influence of body shape on myelomagenesis occurs both at earlier and later ages (12) and is consistent with a recent report from the International Myeloma Cohort Consortium, which found that individuals who were obese both in earlier and later adulthood had the greatest risk of MM (12). Similar trends were observed in joint analyses of obesity in earlier and later adulthood in two other reports (11,13) but interestingly were not replicated in our cohorts (10). However, none of the previous reports included measures before young adulthood.

Central adiposity has been inconsistently associated with MM. A study of MM mortality in 1.5 million participants pooled from 20 cohorts (n = 1388 MM deaths) found a positive association for waist circumference and a suggestive positive association for WHR (13). However, two earlier cohort studies with limited case numbers observed no statistically significant association for waist circumference or WHR with lymphoproliferative cancers (combined) or MM (16,18). The current analysis found no statistically significant associations between central adiposity, as assessed by waist circumference and WHR, and MM risk. Our observation that a larger hip circumference was associated with increased MM risk is consistent with an earlier prospective study of 37 083 postmenopausal women (n = 95 MM cases) that reported a statistically significant trend for increased MM risk across tertiles of hip circumference (15). Although this association is intriguing, the mechanistic interpretation is unclear, and several other investigations observed no association of hip circumference with MM risk (14,16,18).

Limitations include the fact that our primary exposure variables are based on self-reported data and, although prospectively assessed and validated (22,25), are subject to misclassification. We queried waist and hip circumference on only a few questionnaires per cohort and had to exclude the first 10 years of follow-up from those analyses in the NHS. Thus, misclassification of those variables and limited statistical power may have prevented detection of an association with MM. We may also have overestimated the number of individuals with stable weight in the weight pattern analysis, given that our calculations were based on 11- and 16-year net weight change instead of the full 20-year exposure period. Additionally, although the group-based trajectory modeling approach is a strength, the derived trajectories may not accurately reflect an individual’s unique change in body shape. However, we used stringent criteria (30) during trajectory selection to ensure the patterns we identified adequately described the sample. Further, our study populations were homogeneous—comprising predominantly white individuals in health-related occupations—and thus our findings may not be generalizable to other nonwhite populations; however, the association of obesity with MM risk does not appear to differ by race or other demographics (9,12). Study strengths include the prospective design, validated and frequently updated exposure information, long follow-up periods, and sufficient cases of MM for well-powered analyses. The trajectory modeling of body shape through age 60 years also allowed for modeling of multiple body shape exposures that are easy to interpret.

Our results suggest that a larger and increasing body shape through age 60 years and possibly extreme weight cycling and peripheral adiposity are modifiable risk factors for MM that warrant confirmation in other well-powered prospective studies. Collectively, our findings support the notion that avoiding weight gain by maintaining a lean and stable weight throughout life, particularly beginning early in life—in keeping with public health recommendations—confers the added benefit of MM prevention.

## Funding

This study was funded in part by the National Institutes of Health (K07 CA115687, R01 CA127435, P01 CA87969, UM1 CA186107, UM1 CA167552, R01 CA149445, R21 CA198239, F32 CA220859, K99 CA215314, R03 CA204825) and the American Cancer Society (MRSG-17–220-01–NEC, RSG-11–020-01-CNE, PF-17–231-01-CCE, and Clinical Research Professorship [GAC]), and institutional funds from the Dana-Farber Cancer Institute. This research was also supported by a Stand Up To Cancer Dream Team Research Grant (grant number: SU2C-AACR-DT-28-18). Stand Up To Cancer is a program of the Entertainment Industry Foundation. Research grants are administered by the American Association for Cancer Research, the scientific partner of Stand Up To Cancer. Opinions, interpretations, conclusions, and recommendations are those of the authors and are not necessarily endorsed by Stand Up To Cancer, the Entertainment Industry Foundation, or the American Association for Cancer Research.

The funding sources had no role in the design, collection, analysis, interpretation or reporting of the study described herein, or in the decision to submit for publication.

## Notes

Affiliations of authors: Division of Population Sciences, Department of Medical Oncology, Dana-Farber Cancer Institute, Boston, MA (CRM, TRR); Department of Epidemiology, Harvard T.H. Chan School of Public Health, Boston, MA (CRM, EG, MS, TRR, GAC); Department of Nutrition, Harvard T.H. School of Public Health, Boston, MA (EG, MS); Channing Division of Network Medicine, Department of Medicine, Brigham and Women’s Hospital and Harvard Medical School, Boston, MA (CAS, EG, BAR, BMB); Clinical and Translational Epidemiology Unit and Division of Gastroenterology, Massachusetts General Hospital, Boston, MA (MS); Department of Nutrition, Institute of Basic Medical Sciences, University of Oslo, Oslo, Norway (ASK); Department of Cancer Epidemiology, Moffitt Cancer Center, Tampa, FL (MKT); Department of Surgery and Alvin J. Siteman Cancer Center, Washington University School of Medicine, and Barnes Jewish Hospital, St. Louis, MO (GAC).

The authors declare no conflicts of interest.

Lastly, we would like to thank the participants and staff of the Health Professionals Follow-Up Study and Nurses’ Health Study for their valuable contributions as well as the following state cancer registries for their help: AL, AZ, AR, CA, CO, CT, DE, FL, GA, ID, IL, IN, IA, KY, LA, ME, MD, MA, MI, NE, NH, NJ, NY, NC, ND, OH, OK, OR, PA, RI, SC, TN, TX, VA, WA, and WY. The authors assume full responsibility for analyses and interpretation of these data.

## Supplementary Material

pkz044_Supplementary_Data
