# Supplementary material for: Elucidating Under-Studied Aspects of the Link Between Obesity and Multiple Myeloma: Weight Pattern, Body Shape Trajectory, and Body Fat Distribution
Source: JNCI Cancer Spectr. 2019 Jun 24;3(3):pkz044. doi: 10.1093/jncics/pkz044 (PMC6699596; doi:10.1093/jncics/pkz044)
Supplement: pkz044_Supplementary_Data [file pkz044_supplementary_data.pdf]

Supplementary Material: Elucidating under-studied aspects of the link between obesity and multiple myeloma: weight pattern, body shape trajectory, and body fat distribution

JNCI Cancer Spectrum

Supplementary Methods:

<u>Categorization of somatotype category from body mass index (BMI; for use in body shape trajectory estimation)</u>
The oldest age for which we had somatotype data was age 40 years. To facilitate estimation of body shape trajectories through age 60, we divided BMI at ages 45, 50, 55, and 60 into nine categories, consistent with the nine levels of somatotypes queried at younger ages. The upper cut-points for each category were calculated as the median BMI of the given category at age 40 plus a constant to account for weight gain from age 40 to the age of interest (e.g., 40 to 45, 40 to 50, 40 to 55, or 40 to 60). For example, in women in the Nurses' Health Study (NHS), the median BMI at age 40 for the first (leanest) somatotype category was 19.3, and the mean increment of BMI change between age 40 to 50 was 0.8 kg/m^2. Therefore, the upper cutoff for the first somatotype category at age 45 was 19.3+0.8=20.1, as shown below. Cut-points were determined similarly for the other categories in NHS and for men in the Health Professionals Follow-up Study (HPFS). A full list of the cutoffs used to derive somatotype categories at age 45, 50, 55, and 60 in NHS and HPFS are provided below.

<u>NOTE:</u> data below denote the BMI ranges for each somatotype category in the cohorts

<u>NHS:</u>
Age 45 BMI:
Somatotype 1: <=20.1
Somatotype 2: >20.1 and <=20.8
Somatotype 3: >20.8 and <=22.1
Somatotype 4: >22.1 and <=23.8
Somatotype 5: >23.8 and <=26.5
Somatotype 6: >26.5 and <=30.0
Somatotype 7: >30.0 and <=34.1
Somatotype 8: >34.1 and <=37.9
Somatotype 9: >37.9

Age 50 BMI:
Somatotype 1: <=20.8
Somatotype 2: >20.8 and <=21.5
Somatotype 3: >21.5 and <=22.8
Somatotype 4: >22.8 and <=24.5
Somatotype 5: >24.5 and <=27.2
Somatotype 6: >27.2 and <=30.7
Somatotype 7: >30.7 and <=34.8
Somatotype 8: >34.8 and <=38.6
Somatotype 9: >38.6

Age 55 BMI:
Somatotype 1: <=21.4
Somatotype 2: >21.4 and <=22.1
Somatotype 3: >22.1 and <=23.4
Somatotype 4: >23.4 and <=25.1
Somatotype 5: >25.1 and <=27.8
Somatotype 6: >27.8 and <=31.3
Somatotype 7: >31.3 and <=35.4
Somatotype 8: >35.4 and <=39.2
Somatotype 9: >39.2

Age 60 BMI:
Somatotype 1: <=22.0
Somatotype 2: >22.0 and <=22.7
Somatotype 3: >22.7 and <=24.0
Somatotype 4: >24.0 and <=25.7
Somatotype 5: >25.7 and <=28.4
Somatotype 6: >28.4 and <=31.9
Somatotype 7: >31.9 and <=36.0
Somatotype 8: >36.0 and <=39.8
Somatotype 9: >39.8

HPFS:
Age 45 BMI:
Somatotype 1: <=20.7
Somatotype 2: >20.7 and <=22.0
Somatotype 3: >22.0 and <=23.0
Somatotype 4: >23.0 and <=24.2
Somatotype 5: >24.2 and <=25.9
Somatotype 6: >25.9 and <=28.3
Somatotype 7: >28.3 and <=31.7
Somatotype 8: >31.7 and <=35.6
Somatotype 9: >35.6

Age 50 BMI:
Somatotype 1: <=21.1
Somatotype 2: >21.1 and <=22.4
Somatotype 3: >22.4 and <=23.4
Somatotype 4: >23.4 and <=24.6
Somatotype 5: >24.6 and <=26.3
Somatotype 6: >26.3 and <=28.7
Somatotype 7: >28.7 and <=32.1
Somatotype 8: >32.1and <=36.0
Somatotype 9: >36.0

Age 55 BMI:
Somatotype 1: <=21.4
Somatotype 2: >21.4 and <=22.7
Somatotype 3: >22.7 and <=23.7
Somatotype 4: >23.7 and <=24.9
Somatotype 5: >24.9 and <=26.6
Somatotype 6: >26.6 and <=29.0
Somatotype 7: >29.0 and <=32.4
Somatotype 8: >32.4 and <=36.3
Somatotype 9: >36.3

Age 60 BMI:
Somatotype 1: <=21.5
Somatotype 2: >21.5 and <=22.8
Somatotype 3: >22.8 and <=23.8
Somatotype 4: >23.8 and <=25.0
Somatotype 5: >25.0 and <=26.7
Somatotype 6: >26.7 and <=29.1
Somatotype 7: >29.1 and <=32.5
Somatotype 8: >32.5 and <=36.4
Somatotype 9: >36.4

Supplement Table 1. Age-Standardized Characteristics[a] of Participants Enrolled in the Nurses' Health Study (NHS) and Health Professionals Follow-up Study (HPFS) Stratified by Categories of Weight Cycling.

	Weight Loss N = 3906 (3.8%)	Weight Maintenance N = 7360 (7.2%)	Weight Gain N = 13986 (13.7%)	Light Cycling N = 26767 (26.3%)	Moderate Cycling N = 25739 (25.3%)	Extreme Cycling N = 24122 (23.7%)
Age at enrollment, years[b]	53.5 (9.8)	50.8 (9.8)	45.9 (9.0)	46.0 (9.3)	44.8 (9.0)	43.3 (8.4)
Race, %						
White, %	95	94	96	96	96	97
Black, %	1	1	1	1	1	1
Asian, %	2	2	1	1	1	0
Other/missing, %	3	3	2	2	2	2
BMI at enrollment, kg/m^2						
NHS	23.0 (4.2)	21.0 (2.4)	21.5 (2.8)	22.3 (2.8)	23.8 (3.5)	26.5 (4.7)
HPFS	24.0 (2.8)	23.5 (2.3)	24.2 (2.6)	25.0 (2.3)	26.2 (2.7)	27.6 (4.0)
Young adult BMI, kg/m^2						
NHS	21.0 (2.8)	20.2 (2.2)	20.2 (2.4)	20.7 (2.4)	21.4 (2.7)	22.7 (3.5)
HPFS	22.5 (2.6)	22.0 (2.3)	21.7 (2.3)	22.6 (2.4)	23.5 (2.7)	24.6 (3.4)
BMI at age 40						
NHS	21.9 (6.0)	20.4 (2.6)	21.4 (2.8)	22.1 (2.7)	23.8 (3.3)	27.0 (4.7)
HPFS	23.5 (4.4)	22.9 (2.3)	23.5 (2.3)	24.5 (2.3)	25.5 (2.4)	27.2 (3.4)
BMI at age 50						
NHS	22.0 (4.4)	21.0 (2.5)	23.0 (3.3)	23.6 (3.2)	25.6 (3.9)	28.9 (5.3)
HPFS	23.4 (3.6)	23.2 (2.4)	24.6 (2.5)	25.2 (2.4)	26.5 (2.8)	28.2 (3.8)
BMI at age 60						
NHS	21.9 (4.3)	21.5 (2.6)	24.3 (3.7)	24.8 (3.6)	27.0 (4.3)	30.4 (5.7)
HPFS	23.7 (3.1)	23.7 (2.3)	25.2 (2.9)	25.7 (2.6)	27.1 (3.0)	28.7 (4.2)

Abbreviations: BMI: Body Mass Index
[a]Values are means(SD) or percentages and are standardized to the age distribution of the study population; values of polytomous variables may not sum to 100% due to rounding.
[b]Value is not age standardized.

Supplement Table 2. Joint Categories of Baseline BMI and 20-Year Weight Pattern and the Risk of Multiple Myeloma Among Participants in the Nurses' Health Study (NHS) and Health Professionals Follow-Up Study (HPFS).

	NHS			HPFS			Pooled[a,b]
	**Cases**	**Person-Years**	**HR (95% CI)[c]**	**Cases**	**Person-Years**	**HR (95% CI)[c]**	**HR (95% CI)[c]**
**Model 1: Adjusted for age, follow-up period and height**							
No Cycling, BMI <25	38	451282	1	25	136792	1	1
No Cycling, BMI ≥25	7	50452	1.44 (0.64 to 3.24)	13	58539	1.02 (0.52 to 2.03)	1.19 (0.71 to 1.99)
Cycler, BMI <25	112	1286615	1.11 (0.76 to 1.61)	30	168001	1.23 (0.72 to 2.12)	1.12 (0.83 to 1.52)
Cycler, BMI ≥25	60	561271	1.26 (0.84 to 1.90)	53	266216	1.21 (0.74 to 1.96)	1.22 (0.90 to 1.67)

Abbreviations: HR: Hazard Ratio; CI: Confidence Interval; BMI: Body Mass Index

[a]P-values for heterogeneity by sex (cohort) were all ≥0.53.

[b]Pooled models additionally adjusted for sex (cohort).

[c]HRs and 95% CIs were calculated in Cox proportional hazard models stratified on age and calendar period of follow-up.

Supplement Table 3. Age-Standardized Characteristics[a] of Participants Enrolled in the Nurses' Health Study (NHS) and Health Professionals Follow-up Study (HPFS) Stratified by Category of Body Shape Trajectory.

	**Lean-stable N = 30380 (20.4%)**	**Lean-increase N = 42032 (28.3%)**	**Medium-stable N = 42643 (28.7%)**	**Medium-increase N = 33650 (22.6%)**
Age at enrollment, years[b]	47.1 (9.3)	47.5 (9.6)	44.7 (8.7)	43.0 (8.5)
Race, %				
White, %	95	94	96	95
Black, %	1	2	1	2
Asian, %	2	1	1	0
Other/missing, %	2	3	1	2
BMI at enrollment, kg/m²				
NHS	20.0 (1.4)	23.3 (2.5)	22.8 (2.0)	28.8 (4.7)
HPFS	22.7 (1.9)	26.0 (2.3)	24.1 (1.9)	28.7 (3.6)
Young adult BMI, kg/m²				
NHS	19.8 (2.0)	20.2 (2.2)	21.5 (2.4)	23.7 (3.6)
HPFS	20.9 (1.9)	22.9 (2.4)	22.9 (2.1)	25.8 (3.1)
BMI at age 40				
NHS	19.9 (1.4)	22.9 (2.2)	22.5 (1.8)	28.8 (4.3)
HPFS	21.9 (1.5)	25.2 (1.9)	23.4 (1.4)	28.4 (2.9)
BMI at age 50				
NHS	20.5 (1.3)	24.9 (2.4)	24.0 (1.8)	31.7 (4.5)
HPFS	22.3 (1.4)	26.4 (1.9)	23.9 (1.3)	29.9 (3.3)
BMI at age 60				
NHS	20.9 (1.5)	26.6 (2.8)	25.1 (2.2)	33.3 (4.8)
HPFS	22.8 (1.5)	27.2 (2.3)	24.3 (1.5)	30.4 (3.5)

Abbreviations: BMI: Body Mass Index
[a]Values are means (SD) or percentages and are standardized to the age distribution of the study population; values of polytomous variables may not sum to 100% due to rounding.
[b]Value is not age-standardized.

Supplement Table 4. Adult Body Shape Trajectories (Ages 20-60) and the Risk of Multiple Myeloma Among Participants in the Nurses' Health Study (NHS) and Health Professionals Follow-Up Study (HPFS).

	NHS			HPFS			Pooled[a,b]
	**Cases**	**Person-Years**	**HR (95% CI)[c]**	**Cases**	**Person-Years**	**HR (95% CI)[c]**	**HR (95% CI)[c]**
Lean-stable	43	575068	1	18	110953	1	1
Medium-stable	118	1135621	1.45 (1.02, 2.06)	55	293928	1.17 (0.68, 2.00)	1.34 (0.999, 1.80)
Medium-increase	120	1304620	1.38 (0.97, 1.96)	80	372019	1.46 (0.87, 2.45)	1.42 (1.06, 1.89)
Large-increase	38	381347	1.75 (1.13, 2.73)	25	105396	2.18 (1.17, 4.05)	1.88 (1.32, 2.70)

Abbreviations: HR: Hazard Ratio; CI: Confidence Interval

[a]P-values for heterogeneity by sex (cohort) were all ≥0.50.

[b]Pooled models were additionally adjusted for sex (cohort).

[c]HRs and 95% CIs were calculated in Cox proportional hazard models stratified on age and calendar period of follow-up and adjusted for height.

Supplementary Figure 1a. Trajectories of Body Shape[a] Across Adulthood Among Women in the Nurses' Health Study.

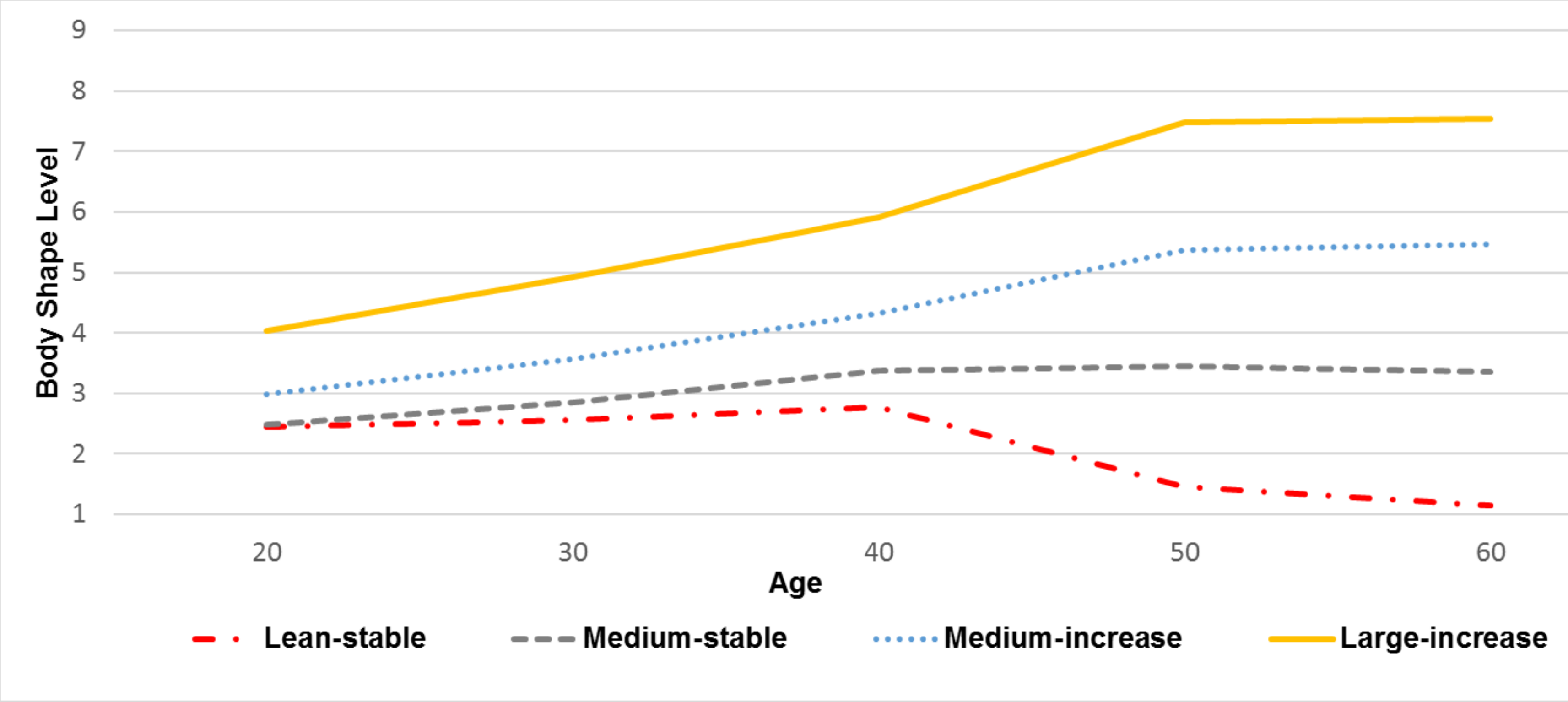

[a]A larger body shape level corresponds to greater body fatness.

Supplementary Figure 1b. Trajectories of Body Shape[a] Across Adulthood Among Men in the Health Professionals Follow-up Study.

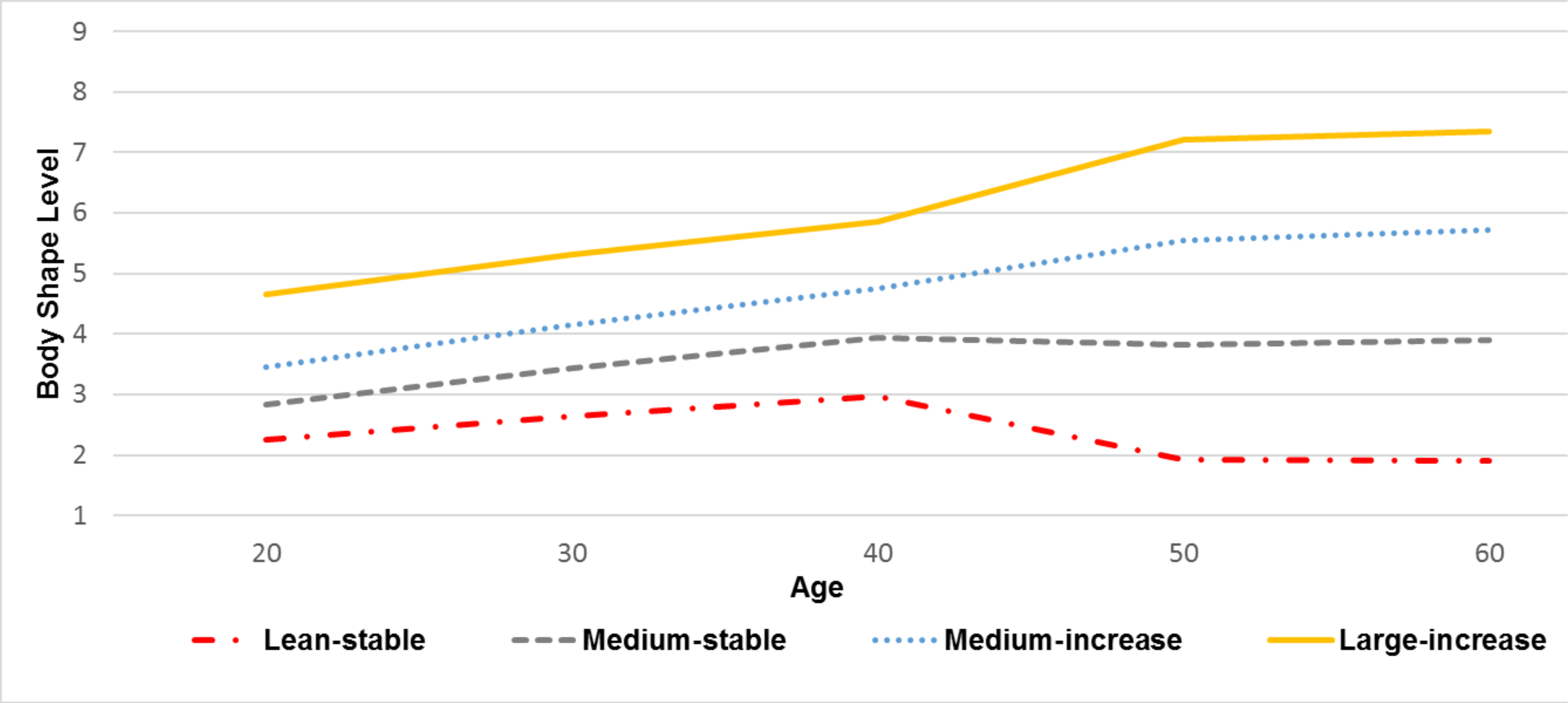

[a]A larger body shape level corresponds to greater body fatness.

Supplement Table 5. Age-Specific Somatotype Reflecting Body Shape and the Risk of Multiple Myeloma Among Participants in the Nurses' Health Study (NHS) and Health Professionals Follow-Up Study (HPFS).

	NHS			HPFS			Pooled[a,b]
	Cases	Person-Years	HR (95% CI)[c]	Cases	Person-Years	HR (95% CI)[c]	HR (95% CI)[c]
**Childhood Somatotype (Average of Ages 5 and 10)**							
1	104	1020779	1	58	263619	1	1
2	53	626789	0.89 (0.64 to 1.24)	33	176865	0.94 (0.61 to 1.45)	0.92 (0.71 to 1.20)
3	46	478288	1.03 (0.72 to 1.46)	36	124440	1.53 (1.00 to 2.33)	1.21 (0.93 to 1.58)
4	32	295524	1.18 (0.79 to 1.76)	15	89179	0.86 (0.47 to 1.55)	1.09 (0.78 to 1.51)
5+	18	183334	1.07 (0.65 to 1.77)	22	97466	1.27 (0.77 to 2.10)	1.18 (0.83 to 1.67)
p-trend[d]			0.47			0.33	0.19
**Adolescent Somatotype (Average of Ages 10 and 20)**							
1	67	683012	1	31	133836	1	1
2	73	820704	0.94 (0.67 to 1.31)	44	243583	0.86 (0.54 to 1.37)	0.91 (0.69 to 1.19)
3	61	606133	1.10 (0.78 to 1.56)	44	170866	1.36 (0.85 to 2.17)	1.18 (0.90 to 1.56)
4	36	366214	1.12 (0.74 to 1.68)	29	116236	1.20 (0.71 to 2.03)	1.18 (0.86 to 1.62)
5+	18	155651	1.29 (0.77 to 2.18)	18	93295	1.19 (0.66 to 2.16)	1.20 (0.81 to 1.77)
p-trend[d]			0.24			0.17	0.08
**Somatotype at Age 30**							
1-2	61	615655	1	14	88152	1	1
3	91	1024631	0.93 (0.67 to 1.29)	37	201763	1.21 (0.65 to 2.26)	0.98 (0.73 to 1.30)
4	72	632340	1.22 (0.86 to 1.72)	61	253353	1.83 (1.01 to 3.30)	1.32 (0.99 to 1.76)
5	19	248079	0.84 (0.50 to 1.40)	38	157104	1.96 (1.05 to 3.65)	1.19 (0.84 to 1.70)
6+	12	106942	1.36 (0.73 to 2.54)	16	55882	2.50 (1.20 to 5.21)	1.62 (1.04 to 2.52)
p-trend[d]			0.41			0.001	0.01
**Somatotype at Age 40**							
1-2	24	267482	1	5	37152	1	1
3	76	786682	1.11 (0.70 to 1.76)	23	122218	1.55 (0.58 to 4.13)	1.16 (0.76 to 1.75)
4	83	855957	1.11 (0.71 to 1.76)	52	248956	1.72 (0.68 to 4.36)	1.19 (0.80 to 1.79)
5	44	451284	1.16 (0.70 to 1.90)	55	249658	1.96 (0.78 to 4.97)	1.28 (0.84 to 1.95)
6+	28	266944	1.35 (0.78 to 2.35)	31	101368	3.04 (1.16 to 7.92)	1.69 (1.08 to 2.66)
p-trend[d]			0.20			0.006	0.01

Abbreviations: HR: Hazard Ratio; CI: Confidence Interval

[a]P-values for heterogeneity by cohort (sex) was p=0.051 for Somatotype at age 30 and ≥0.14 for all other models.

[b]Pooled models additionally adjusted for cohort (sex).

[c]HRs and 95% CIs were calculated in Cox proportional hazard models stratified on age and calendar period of follow-up and adjusted for height.

[d]P-trend: P-value for trend from models treating the somatotype variables as ordinal variables.
